# Economic Policy Uncertainty and Family Firm Innovation: Evidence From Listed Companies in China

**DOI:** 10.3389/fpsyg.2022.901051

**Published:** 2022-06-14

**Authors:** Yong Qi, Shaoyu Dong, Simeng Lyu, Shuo Yang

**Affiliations:** School of Business Administration, Northeastern University, Shenyang, China

**Keywords:** economic policy uncertainty, family firm, innovation, executive background, risk-taking

## Abstract

With the advancement of China’s economic transformation, the impact of economic policy uncertainty on family firms has become increasingly significant. The “familism” of family firms makes them more motivated to maintain family harmony, pursue innovative activities, and the long-term development of enterprises when faced with economic policy uncertainty. In this paper, we employed the data of listed Chinese family firms from 2010 to 2018 to analyze the impact of economic policy uncertainty on family business innovation activities, analyze the inherent characteristics of family firm innovation, and find the path that enables the innovative activities of family firms and provides a valuable experience for the innovation of private enterprises in economic policy uncertainty. We provide evidence that economic policy uncertainty positively relates to family firm innovation. Moreover, the relationship is affected by factors such as directors’ executive background and access to state-owned equity. Further analysis indicates that economic policy uncertainty can promote family firms’ innovation activities by improving their risk-taking, internal capital market circulation, and reducing political connections.

## Introduction

PricewaterhouseCoopers’ ‘Global Family Business Survey 2018 -- China Report’^1^ shows that 55.7% of non-state-owned enterprises in China’s A-share market are family firms. These have already become the most important part of China’s non-state-owned enterprises. This growth is relatively later than that in developed countries, and showing two unique features: (i) shorter survival time, and (ii) more difficulties in firm size growth (Xiang and Li, 2016). As China’s economy enters the ‘new normal’^2^, economic growth is gradually slowing down, and economic policy uncertainty is increasing. Under these conditions, the owners and managers of family firms must change their negative attitudes to resist the restrictions associated with economic policy uncertainty on firm operations. Nevertheless, recent studies have stressed that family firms are facing an innovator’s dilemma characterized by a paradox (Diaz-Moriana et al., 2020; Erdogan et al., 2020). That is, family firms have the ability to innovate more, but they still innovate less. Daniel et al. (2022) focus on what family-specific features affect family firms’ innovation. However, few studies have focused on the impact of economic policy uncertainty on family firms. Focusing on the economic policy uncertainty, this paper analyzes the influence of different characteristics of family firm innovation and their mechanisms, aiming to enrich the empirical research on this topic. As a contribution, this study helps family firms identify whether these characters to innovation are present in their firms, what factors strengthen innovation, and shed light on the underlying mechanism.

Family factors lead family firms to show unique characteristics regarding innovation activities. Some studies show that family members emphasize ensuring control of their firms (Calabrò et al., 2018), so they prefer more conservative financial strategies (Morck and Yeung, 2010), which produces insufficient willingness and resources and further blocks innovation investments (Chen and Wu, 2014). However, some studies argue that family firms prefer long-term return innovation activities (Huang et al., 2018) because of their willingness to undertake sustainable development, which leads family firms to invest positively in innovation. Therefore, research on the innovation behavior of family firms cannot draw conclusions from only one viewpoint. Moreover, previous studies have mainly focused on the effects of internal factors such as ‘innovation ability’ (Massis et al., 2015; Shi et al., 2015; Bauweraerts and Colot, 2017), ‘corporate governance’ (Beck et al., 2011; Deng et al., 2013; Matzler et al., 2015), and ‘family culture’ (Francesco and Salvato, 2014; Wu et al., 2019). However, there is insufficient empirical research on the effects of institutional environmental factors on the innovation behavior of family firms.

Family firms show specific ideological features against a background of institutional environmental factors. Recent studies report a complicated relationship between the institutional environment and family businesses. Ellul et al. (2018) report that unemployment insurance is likely to affect working stability and wages in family firms, and Tsoutsoura (2015) reports the effects of inheritance tax on investment decisions and control transfer of family firms. Facing fluctuations between economic cycles, the Chinese government has implemented a series of economic policies. These economic policies help relieve these fluctuations; however, frequent policy changes increase the uncertainty of economic policies and affect the sensitivity of enterprises to policy and economic factors, and further affect their innovation activities. Therefore, focusing on the stimulus of frequent policy changes to firm innovation activities, and studying the impact of macroeconomic policies on family business innovation activities from the perspective of economic policy uncertainty is of great significance to the long-term development of family firms. However, to the best of our knowledge, few studies have focused on the influence of the institutional environment on family firm innovation.

Economic policy uncertainty represents a typical institutional environment and can affect family firm innovation in several ways. Since policy uncertainty decreases enterprise financialization (Wu et al., 2019) and asset returns (Phan et al., 2019), which reduce firms’ short-term returns, family firms are motivated to increase long-term investment (such as innovation) to maintain smooth operation. Since policy uncertainty increases the costs of external financing (Han and Qiu, 2007) and financial constraints, family firms are motivated to strengthen internal transaction to increase cash holdings. Policy uncertainty can also increase managerial risk-taking, inducing firms to adopt China’s new normal. For these reasons, we expect a positive relationship between economic policy uncertainty and family firm innovation. We begin by examining the effect of economic policy uncertainty on family firm innovation. We use the economic policy uncertainty index developed by Davis et al. (2019)^3^. as a measure of China’s economic policy uncertainty. Using a sample that includes 6,469 observations from 2010 to 2018, we find that economic policy uncertainty is positively related to family firm innovation. Moreover, this relationship is influenced by factors such as executive background and state-owned equity. Further analysis indicates that economic policy uncertainty promotes family firms’ innovation activities by improving their risk-taking ability, internal capital market circulation, and reducing political connections.

Our analysis contributes to the literature on family firm innovation behavior and the effects of economic policy uncertainty studies in several ways. First, we use the economic policy index developed by Davis et al. (2019), which has better continuity and time-varying responses to institutional changes. Previous studies mostly analyze the institutional environment as an exogenous variable and study the influence of non-dynamic changes in the institutional environment. Our research contributes to a burgeoning stream of literature that studies the effects of economic policy uncertainty on corporate behavior.

Second, our study contributes to bridging the existing knowledge gap at the intersection of economic policy uncertainty and family firm innovation. However, the conclusions of previous studies on the innovation behavior of family firms were limited by considering only one viewpoint. Our research aims to clarify the relationship between economic policy uncertainty and family firm innovation, specifically, whether and how different enterprise features moderate this effect. Identifying which factors moderate the relationship between economic policy uncertainty and family firm innovation would allow a better interpretation of the innovation behavior of family firms compared to non-family firms, as well as the heterogeneity among family firms.

Third, previous studies mostly analyze the difference between state and non-state enterprises, which ignores the profound effect of familism on non-state enterprises. Our research focuses particularly on the effects of economic policy uncertainty, which differs from systematic uncertainty in measurement, period, and implications (Hao et al., 2016; Gu et al., 2018).

The rest of this paper is arranged as follows: the second section presents a literature review and the research hypotheses; the third section presents the research design; the fourth section tests the hypotheses using different empirical models; the fifth section presents the discussion of the results obtained; and the last section puts forward the conclusions and implications of our study.

## Literature Review

### Economic Policy Uncertainty

Governments have tended to intervene in the market by issuing economic policies since the 2008 financial crisis and given the profound impact of policy uncertainty on the economy, academic researchers have shown increasing interest in investigating the effects of policy uncertainty on corporate behavior (Phan et al., 2019). However, changes in the macro-environment increase economic policy uncertainty, which has aroused concern in the academic community. Some scholars argue that economic policy uncertainty has negative effects on the macro-economy, and is an important factor for fluctuations in key macroeconomic variables and financial asset variables (Mbanyele, 2020; Wang et al., 2022). Moreover, Baker et al. (2016) find that economic policy uncertainty affects the economic cycle and blocks economic recovery. However, some studies focus on the effect of economic policy uncertainty on micro-enterprise operational activities. Studies show that economic policy uncertainty increases the uncertainty factors faced by enterprises, which affects companies’ investment decisions, fund demand, and business activities by changing business costs (Jin et al., 2014; Wang and Song, 2014; Li and Yang, 2015).

Reviewing the current literature on economic policy uncertainty reveals a contradictory picture. Bhattacharya et al. (2017) argued that uncertainty in national elections weakens enterprises’ innovation motivation, and this effect is more significant for innovation-intensive companies. Hao et al. (2016) argue that economic policy uncertainty delays enterprises’ research and development (R&D) investment decisions and block innovation activities. Chen et al. (2016) focus on the effect of policy uncertainty on enterprise innovation caused by changes in local officials, and argue that economic policy uncertainty will reduce the innovation efficiency of enterprises. More recently, Hudakova et al. (2021) focused on assessing the impact of the length of entrepreneurship on the perception of the most important business risks in V4 countries. The results show that owners and managers of enterprises still consider market and economic risks to be most important in their business. Gu et al. (2018) show that economic policy uncertainty has significant positive effects on the R&D investment and patent applications of enterprises; such positive effects are affected by the nature of the company’s ownership, industry, financial constraints, and government subsidies based on the economic policy uncertainty index by Baker et al. (2016). He et al. (2020) find that economic policy uncertainty is positively correlated with corporate innovation in general. They further conclude that economic policy uncertainty has a stronger positive effect on state-owned enterprises, lower cash flow companies and companies with fewer financial constraints. Guan et al. (2021) find that economic policy uncertainty is positively related to corporate technological innovation, and is negatively related to corporate business model innovation.

In summary, the studies on economic policy uncertainty suggest that the effects of economic policy uncertainty on firms’ innovation activities are significantly heterogeneous. As an important part of private enterprises, the innovation behaviors of family firms are significantly different from those of other non-family enterprises. To the best of our knowledge, none of these studies have focused on the effect of economic policy uncertainty on family firms. Therefore, further exploration will contribute to understanding the relationship between economic policy uncertainty and the innovation activities of family firms.

### Family Firm Innovation

Innovation, as one of the channels essential for a firm to maintain long-term competitive advantage and growth, has received much attention in recent years. Most studies on the micro-influences on family firms’ innovation activities focus on three main levels: individual, family, and firm. Individual-level studies mainly focus on the characteristics of leaders (Sitthipongpanich and Polsiri, 2015), employee loyalty (Chen and Zheng, 2016), and social ties (Yan and Xiao, 2019). Family-level studies mainly focus on family ownership (Ashwin et al., 2015), family control (De Massis et al., 2012), management structure (Memili et al., 2015), intergenerational transition (Akhmedova et al., 2020; Mariotti et al., 2021). Firm-level studies mainly focus on profitability (Chrisman and Patel, 2012), cash flow (Munoz-Bullon and Sanchez-Bueno, 2011), firm age (Akcigit and Kerr, 2010), internationalization (Alayo et al., 2021, 2022) and policy ties (Perchard and Niall, 2020). While many studies have focused on the influence of micro-factors, research on the influence of macroeconomic policy on family firms’ innovation activities remains insufficient.

The macro-environment is an important factor in firm innovation. Hao and Liu (2010) investigate the influence of legal and political environments from the perspective of property rights characteristics. Lv et al. (2017) show that improvements in the formal system will increase the protection of innovations, and family managers will actively promote innovation activities. A recent study by Zhu et al. (2016) finds that an increase in marketization weakens entrepreneurs’ desirability for control and is negatively correlated with the R&D intensity of enterprises. Chen et al. (2018) further show that an increase in marketization will also positively affect the innovation performance of family firms. Yu et al. (2016) find that the impact of industry technology dynamics on innovation and R&D is determined by the length of the family firm’s investment in R&D activities. Lewandowska et al. (2021) examines the impact of financial support for investments, especially from EU Structural Funds, on SMEs competitiveness in Poland. The results shows that SMEs have not used financial support efficiently.

There is a large family business innovation activity literature focusing on inner enterprises at the micro-level, while few studies focus on external factors at the macro-level. Most macro-level studies focus only on the general influence of industrial policy and institutional environment on family firm behavior. However, they do not examine the complex dynamic relationship of China’s institutional environment, large-scale and deep-seated institutional change, and high levels of environmental uncertainty with family firm innovations.

### Hypotheses

Previous studies on innovation activities only focus on the effect of a single type of family firm’s behavior features; however, different behavioral features may have different effects on enterprises’ innovation activities (Ronald et al., 2012).

First, economic policy uncertainty changes the business objectives of family firms. Frequent macroeconomic policy changes bring temporary negative shocks to companies during economic recessions or downturns (Nick et al., 2007), and increase the risk of external investment and financing activities, and shift the business goal from short-term goals (making ‘hot’ money) to long-term goals (survival and sustainable development). In this case, the economic goals of family firms are unified with the goals of social emotional wealth (SEW), which enhances their willingness to engage in innovative activities.

Second, economic policy uncertainty increases risk-taking capacity and changes the innovation activity preferences of family firms. Family firms pay more attention to the continuity and long-term nature of the business because the personal wealth of the family founder is tied to the company. The willingness to take risks and pursue personal achievements will increase family firms’ willingness to take risks and their attention to long-term development opportunities of the company, which is conducive to the family business’s innovative decision-making.

Third, economic policy uncertainty will promote more family resources to support the development of their firms. Family firms pay strong attention to the weakness of control caused by different financing methods, which show significant endogenous financing tendencies in the following order: first, they rely on internal capital; second, they do debt financing; and finally, they consider the external equity capital financing (Geng and Li, 2009). Economic policy uncertainty increases the cost of external financing, where family firms can reduce the first layer of agency costs and further reduce financing costs due to the high concentration of control and management rights and high transparency of information. In addition, a pyramid-shareholding structure enables a family firm to control a large amount of external capital through a small amount of self-owned capital. Desire for business continuity, personal achievement, and long-term business reduces the agency conflict of the second layer and further facilitates the firm’s engagement in innovation activities. The large amount of special capital accumulated by family firms can bring more resource input and reduce the impact of financing constraints on innovation activities.

Based on the above analysis, when facing economic policy uncertainty, the long-term economic goals brought by innovation are consistent with the pursuit of long-term family goals, which enhances the willingness of family firms to innovate. The reduction of agency cost, accumulation of special assets, and family’s emotional attachment to the business provide resource support to invest in innovation activities. Therefore, we propose Hypothesis 1.

*Hypothesis 1:* Economic policy uncertainty leads to positive effects on innovation activities for family firms.

Executives in family firms with financial backgrounds can provide more financial resources through their own social resources. At the same time, as an implicit guarantee of the enterprise, they have a relatively clear understanding of risks and have a high-risk tolerance for corporate financing (Du et al., 2019). Therefore, family firms with financial background executives can reduce the difficulty of financing, help enterprises obtain more loans, and obtain higher credit lines (Deng and Zeng, 2011), which can enable firms to devote more resources to innovation activities. Family firms without financial background executives are more likely to be affected by the uneven development of China’s financial market and imperfect credit system, with limited access to bank loans and can obtain fewer resources for investment in innovation. Therefore, we propose Hypothesis 2.

*Hypothesis 2:* Economic policy uncertainty has more significant promoting effects on innovation activities for family firms with financial background executives compared with other family firms.

Executives with overseas backgrounds are more likely to have advanced technology and management experience, and their thinking methods and management concepts are more international and market-oriented. Therefore, they may tend to have higher corporate strategic positioning and are more inclined to increase investment in research and development. Compared with other family businesses, the overseas background of executives promotes innovation in family firms (Liu et al., 2017). At the same time, domestic and foreign dual social networks can establish international relationships and provide convenient conditions for family firms’ internationalization. Lee and Park (2008) argue that the overseas experience of management teams is conducive to international diversification and can promote the formation of international alliances. Therefore, these firms commonly have advanced innovation activities and strong competitive advantages, which can withstand the impact of uncertainty on enterprises. Therefore, we propose Hypothesis 3.

*Hypothesis 3:* Compared with other family firms, economic policy uncertainty has a stronger promoting effect on innovation activities when executives do not have overseas backgrounds.

Family firms with state-owned equity participation have stronger risk-taking capacities. In addition, governments at all levels are the actual controllers of state-owned equity as well as the main distributor of economic resources, and usually have a certain resource preference for enterprises with state-owned equity participation (Zhang and Zhang, 2016). When state-owned equity participates in family firms, these family firms will also obtain such skewed resources (Yu et al., 2017), which alleviates the financing constraints of enterprises when facing economic policy uncertainty. Therefore, family firms with state-owned equity participation are more willing to innovate, have more innovation resources, and have less impact on them from economic policy uncertainty. In summary, we propose Hypothesis 4.

*Hypothesis 4:* Economic policy uncertainty has a more significant promoting effect on innovation activities for family firms without state-owned equity participation.

## Methodology

### Sample

This study examines the family firms listed in the Board of A Stock Exchange from 2010 to 2018, which were gathered from the China Stock Market and Accounting Research (CSMAR) and Wind databases. The financial data, corporate governance data, and registration place of listed companies are mainly from the CSMAR database. Investment in innovation data come from the Wind database. To identify the family firms, we use the following two criteria: the firms are directly or indirectly controlled by an individual or family-owned entity, and the number (more than one) of entrepreneurs/executives (including middle management) related to the family that owns the firm. Otherwise, the initial samples are deleted according to the following criteria: (1) the industry of financial and insurance listed companies, and (2) ST, ST*, and firms with abnormal transaction status. We obtained a final sample of 6,469 Chinese family firms.

#### Dependent Variable

We assess innovation investment using a continuous variable expressed as a percentage and obtained by dividing the investment in R&D by the operating revenue in the given year. Furthermore, we assessed the natural logarithm of R&D investment to check for robustness.

#### Independent Variables

We use the economic policy uncertainty measure (*EPU*_*M*_) constructed by Davis et al. (2019)^4^. They quantify uncertainty-related concepts from October 1949 onwards using two mainland Chinese newspapers: the Renmin Daily and the Guangming Daily. To construct an economic policy uncertainty index for China, they first obtain monthly counts of articles that contain at least one term in each of three term sets: economics, policy, and uncertainty. In the second step, they scale the raw monthly economic policy uncertainty index counts by the total number of articles for the same newspaper and month. In our analysis, we construct an economic policy uncertainty measure as the weighted arithmetic average of economic policy uncertainty index values in a given year.

#### Feature Variables

‘*Overseas*’ is a dummy variable that takes a value of 1 if at least one of the directors or supervisors or senior management in a firm has overseas study or employment experience, and 0 otherwise. ‘Financial background’(*FB*) is a dummy variable that takes a value of 1 if at least one of the directors, supervisors, or senior management in a firm has employment experience in policy banks, commercial banks, insurance companies, securities companies, fund management companies, futures companies, investment banks, trust companies, investment management companies, or exchanges, and 0 otherwise. ‘*State*’ is also a dummy variable that takes a value of 1 if a family firm has state-owned equity participation, and 0 otherwise.

#### Control Variables

Following the mainstream literature on family firms, we introduce several control variables into our analysis to control for firm characteristics. ‘*Size*’ is the natural logarithm of the book value of total assets. Return on assets (*Roa*) is the operating income, scaled by total assets. ‘*Age*’ is the number of years between the listing date and established date. Because corporate governance mechanisms can also influence family firms’ innovation, we include proxies for various governance devices. ‘*Lev*’ is the total liabilities divided by total assets. ‘*Cashflow*’ refers to the cash flow from a company’s operations and activities divided by total assets. ‘*Tq*’ is the market value of a company’s equity divided by its total assets. ‘*Growth*’ is the difference between the company’s current year’s operating income and the company’s period of last year’s operating income divided by the company’s period of last year’s operating income. ‘*Diver*’ refers to the number of a company’s main businesses. ‘*IndeDir*’ is the number of independent directors divided by the board size. ‘*FamDir*’ is the number of family directors of a company divided by board size. ‘*FamHold*’ is the shareholding ratio of controlling shareholders. ‘*Separation*’ is the actual controller ownership ratio divided by the control ratio. ‘*HHI*’ is the sum of squares of results where the company’s main business revenue in the industry is divided by the industry’s main business revenue. In addition, we also set dummy variables *industry* and *year* in our regression.

### Empirical Model

In this paper, we adopt the panel fixed effect model to analyze and test the effects of economic policy uncertainty on family firm innovation activities. We represent the following regression model (1) to test Hypothesis 1.


(1) $$R\,D_{i,t}=a_{0}+a_{1}\,E\,P\,U_{M,t}+a_{2}\,C\,o\,n\,t\,r\,o\,l_{i,t}+\varepsilon_{i,t}$$

In our model, *i* refers to the *i^th^* family firm, and *t* refers to the year, dependent variable *RD*_*i,t*_ refers to the R&D investment of the *i^th^* individual family firm in year *t*. *EPU*_*M,t*_ refers to the economic policy uncertainty in year *t*. *Control*_*i,t*_ refers to the control variables of the *i^th^* family firm in year *t*, and ε_i,t_ is the residual term.

We represent the regression model (2) to test Hypothesis 2, 3, and 4 as follows.


(2) $$R\,D_{i,t}=\alpha_{0}+\alpha_{1}\,E\,P\,U_{M,t}+\alpha_{2}\,C\,o\,n\,t\,r\,o\,l_{i,t}+a_{3}\,E\,P\,U_{M,t}*X_{i,t}+\varepsilon_{i,t}$$

In this model, *X*_*i,t*_ is a dummy variable of executive feature, which takes a value from *FB*_*i,t*_, Overseas_*i,t*_., or *State*_*i,t*_. When testing Hypothesis 2, *X*_*i,t*_ takes value from *FB*_*i,t*_; the value of *FB*_*i,t*_ is 1 if the executives of the *i^th^* family firm in year *t* possess a financial background, and 0 otherwise. When test Hypothesis 3, *X*_*i,t*_ takes value from *Overseas*_*i,t*_; the value of *Overseas*_*i,t*_ is 1 if the executives of the ith family firm in year *t* possess overseas experience, and 0 otherwise. When testing Hypothesis 4, *X*_*i,t*_ takes value from *State*_*i,t*_; the value of *State*_*i,t*_ is 1 if the executives of the *i^th^* family firm in year *t* hold state-owned equity participation, and 0 otherwise, and ε_i,t_ is the residual term.

## Results

Before the empirical analysis, in order to ensure the heterogeneity and effectiveness of the models, we winsorized the main continuous variables in quantiles of 1 and 99%. Fixed-effects regression was used to control for endogeneity measures in the models as well as industry and year. All data processing was conducted using Stata 15.0 software.

### Descriptive Statistics

Table 1 illustrates the descriptive statistics. The sample average R&D ratio is 5.3%, which indicates that family firms have less investment in innovation. The sample average *Overseas* ratio is 5.7%. The sample average *FB* ratio is 77.9%. The sample average *state* ratio is 11.1%. The *EPU*_*M*_ is 1.381, the maximum *EPU*_*M*_ is 2.778, and the minimum *EPU*_*M*_ is 0.921, which indicates that economic policy uncertainty is highly uncertain and volatile in China over the last 10 years.

**TABLE 1:** Descriptive statistics of variables.

Variable	Obs	Mean	Std. dev.	Min	Max
EPU_*M*_	6469	1.381	0.569	0.921	2.778
RD	6469	0.053	0.115	0	0.759
Lev	6469	0.166	0.226	0	1.685
Roa	6469	0.043	0.076	−2.33	1.722
Tq	6469	2.074	1.184	0.952	7.76
Growth	6469	1.132	8.237	−19.831	63.483
Size	6469	21.527	1.105	18.473	26.237
Age	6469	2.551	0.481	0	3.738
FamDir	6469	0.226	0.114	0	0.667
CashFlow	6469	0.293	0.927	−16.34	13.56
Diver	6469	2.083	1.612	0	12
HHI	6469	0.099	0.121	0	1
FamHold	6469	11.284	16.499	0	70.707
Seperation	6469	0.634	0.394	0	1
Political	6469	0.271	0.445	0	1
Overseas	6469	0.057	0.232	0	1
FB	6469	0.779	0.415	0	1
IndeDir	6469	0.383	0.094	0.125	1
EPU	6469	2.352	1.195	0.989	4.605
EPU_G	6469	0.014	0.003	0.011	0.019
LnRD	6469	0.023	0.021	0	0.112
ICM	6469	0.041	0.342	−0.004	10.684
State	6469	0.111	0.299	1	0

This table shows the descriptive statistics of the variables, *EPU*_*M*_ is the annual average economic policy uncertainty divide by 100; *RD* is the enterprises’ research and development; *Lev* is the total liabilities divided by total assets; *Roa* is the operating income; *Tq* is the market value of a company’s equity divided by its total assets; *Growth* is the difference between the company’s current year’s operating income and the company’s period of last year’s operating income divided by the company’s period of last year’s operating income; *Size* is the natural logarithm of the book value of total assets; *Age* is the number of years between the listing date and established date; *FamDir* is the number of family directors of a company divided by board size; *Cashflow* refers to the cash flow from a company’s operations and activities divided by total assets; *Diver* refers to the number of a company’s main businesses; *HHI* is the sum of squares of results where the company’s main business revenue in the industry is divided by the industry’s main business revenue; *FamHold* is the shareholding ratio of controlling shareholders; *Separation* is the actual controller ownership ratio divided by the control ratio; *FB* is a dummy variable that takes a value of 1 if at least one of the directors, supervisors, or senior management in a firm has employment experience in policy banks, commercial banks, insurance companies, securities companies, fund management companies, futures companies, investment banks, trust companies, investment management companies, or exchanges, and 0 otherwise; *IndeDir* is the number of independent directors divided by the board size; *EPU* is the economic policy uncertainty index by Baker et al. (2016); *EPU_G* is the annual arithmetic of the global economic policy uncertainty by Baker et al. (2016); *LnRD* is the logarithm of R&D; *ICM* refers to the internal capital markets; *State* is also a dummy variable that takes a value of 1 if a family firm has state-owned equity participation, and 0 otherwise; Political is a dummy variable that takes a value of 1 if at least one of the directors, supervisors, or senior management in a firm has employment experience in the Central Committee of the Communist Party of China, the National People’s Congress, the Chinese People’s Political Consultative Conference, the National Party Congress, local people’s governments at all levels, institutional departments, or local committees of the Communist Party of China, and 0 otherwise.

### Baseline Regressions

(1) Economic policy uncertainty and family business innovation investment.

Table 2 reports the results of investment in family firm innovation. Consistent with our expectation, column 1 indicates that the coefficients of economic policy uncertainty are positive (0.0127 and 0.0456), and statistically significant at the 1% level. To test the robustness of our model, we performed an analysis using the logarithm of R&D investment in column 2. This also presents a significant relationship. Our calculation indicates that, although innovation is a long-term investment with high investment and high risk, family enterprises invest less in innovation because of their own ownership. However, the goal of family firms changes from maintenance of firm control to protection of survival and continuity, which improves the willingness to innovate during economic policy uncertainty.

**TABLE 2:** Basic regression.

Variable	(1)	(2)
	RD	LnRD
EPU_*M*_	0.0456***	0.0127***
	(3.9748)	(7.3834)
Lev	−0.0015**	−0.0000
	(−2.4622)	(−0.2037)
Roa	0.0322*	0.0129***
	(1.6904)	(3.2086)
Tq	−0.0048***	−0.0007**
	(−3.4829)	(−2.2143)
Growth	0.0003**	0.0000
	(2.0042)	(1.4635)
Size	0.0267***	−0.0109***
	(4.4132)	(−10.4812)
Age	−0.0159	−0.0037
	(−1.0164)	(−1.2963)
Diver	−0.0021	−0.0002
	(−1.0878)	(−0.6543)
HHI	−0.0223	−0.0048
	(−0.5273)	(−0.8610)
FamHold	0.0003*	0.0000
	(1.7183)	(1.5570)
Seperation	−0.0029	−0.0025
	(−0.2577)	(−1.4604)
CashFlow	−0.0008	0.0009**
	(−0.5873)	(2.3081)
_cons	−0.5405***	0.2341***
	(−4.3278)	(11.2095)
Year	Y	Y
Industry	Y	Y
*N*	6469	6469
adj. *R*^2^	0.1359	0.1311

***, **, and * denote significance at 1, 5, and 10% levels.

(2) Enterprise heterogeneity.

Table 3 indicates that the estimated coefficient of non-overseas is positive (0.0536) and statistically significant at the 1% level in column 2. On the contrary, this influence is not significant in executives with overseas backgrounds in column 1. Our calculation indicates that non-overseas executives do not have the support of social capital from overseas as a risk hedge, which improves awareness of positively resisting risks and enhances risk-taking. The pursuit of enterprise continuity encourages them to carry out innovation activities actively.

**TABLE 3:** Group inspection of family firm’s executives with overseas background.

Variable	(1)	(2)
	(Overseas = 1)	(Overseas = 0)
EPU_*M*_	0.1016	0.0536***
	(0.6232)	(4.5315)
Lev	0.0008	−0.0016**
	(1.2043)	(−2.3656)
Roa	−0.0464	0.0295
hows that, compared wit	(−0.9411)	(1.5229)
Tq	−0.0092***	−0.0044***
	(−3.1974)	(−3.1925)
Growth	−0.0002	0.0003**
	(−0.8934)	(2.1738)
Size	0.0009	0.0260***
	(0.1300)	(4.1430)
Age	0.0439	−0.0246
	(0.8676)	(−1.6357)
Diver	0.0002	−0.0022
	(0.1035)	(−1.1175)
HHI	−0.0024	−0.0198
	(−0.0546)	(−0.5355)
FamHold	0.0002	0.0004**
	(0.5636)	(2.0157)
Seperation	0.0378	−0.0005
	(0.9886)	(−0.0417)
CashFlow	−0.0061*	−0.0007
	(−1.9319)	(−0.5094)
_cons	−0.2188	−0.4772***
	(−1.6463)	(−3.9077)
Year	Y	Y
Industry	Y	Y
*N*	370	6099
adj. *R*^2^	0.2278	0.1347

***, **, and * denote significance at 1, 5, and 10% levels.

Table 4 indicates that the estimated coefficient of financial background is positive (0.0532) and statistically significant at the 1% level in column 2. On the contrary, this influence is not significant in executives with non-financial backgrounds in column 1. Our calculation indicates that executives with financial backgrounds can receive financial assistance and alleviate external financial constraints, which improves the resources of innovation activities.

**TABLE 4:** Group inspection of family firm’s executives with financial background.

Variable	(1)	(2)
	(FB = 0)	(FB = 1)
EPU_*M*_	0.0298	0.0532***
	(1.5970)	(3.6619)
Lev	−0.0008	−0.0016**
	(−0.6432)	(−2.2251)
Roa	0.0077	0.0386
	(0.3958)	(1.2938)
Tq	−0.0002	−0.0053***
	(−0.0961)	(−3.1944)
Growth	−0.0000	0.0003**
	(−0.1112)	(2.0186)
Size	0.0351***	0.0266***
	(2.6023)	(3.7954)
Age	−0.0617	−0.0168
	(−1.4669)	(−0.9560)
Diver	0.0068*	−0.0046*
	(1.9412)	(−1.8989)
HHI	−0.0681	−0.0199
	(−1.2010)	(−0.3953)
FamHold	−0.0000	0.0004*
	(−0.0018)	(1.8231)
Seperation	−0.0070	−0.0034
	(−0.3779)	(−0.2444)
CashFlow	−0.0037	−0.0007
	(−1.0806)	(−0.4535)
_cons	−0.6934**	−0.4684***
	(−2.5482)	(−3.3626)
Year	Y	Y
Industry	Y	Y
*N*	1430	5039
adj. *R*^2^	0.1490	0.1444

***, **, and * denote significance at 1, 5, and 10% levels.

Table 5 indicates that the estimated coefficient of non-state-owned equity is positive (0.0492) and statistically significant at the 1% level in column 2. On the contrary, this influence is not significant in firms that have state-owned equity in column 1. Our calculation indicates that family firms’ non-state-owned equity should rely on their own strength to resist environmental uncertainty. Managers pay more attention to the survival and long-term development of enterprises by actively participating in innovation activities to improve their competitiveness of the enterprise.

**TABLE 5:** Group inspection of state-owned equity participation in family firm.

Variable	(1)	(2)
	State = 1	State = 0
EPU	0.1665	0.0492***
	(1.1013)	(3.9915)
Lev	−0.0008	−0.0012**
	(−0.4461)	(−2.1052)
Roa	−0.0185	0.0326
	(−0.6512)	(1.2905)
Tq	−0.0033	−0.0054***
	(−0.5599)	(−3.3067)
Growth	0.0008	0.0003*
	(1.3469)	(1.9147)
Size	0.0474*	0.0232***
	(1.8523)	(4.0909)
Age	−0.0518	−0.0145
	(−1.1914)	(−0.8875)
Diver	0.0023	−0.0021
	(0.8755)	(−1.0016)
HHI	−0.1137	−0.0148
	(−1.5097)	(−0.3308)
FamHold	0.0003	0.0003
	(0.7004)	(1.4690)
Seperation	0.0136	−0.0031
	(0.4327)	(−0.2921)
CashFlow	0.0176	−0.0010
	(1.3084)	(−0.6399)
_cons	−1.0294**	−0.4776***
	(−2.0417)	(−3.9812)
Year	Y	Y
Industry	Y	Y
*N*	644	5825
adj. *R*^2^	0.0952	0.1384

***, **, and * denote significance at 1, 5, and 10% levels.

### Endogeneity Issue and Robustness Test

We conduct the following robustness tests to ensure the reliability of the main conclusions:

(1) Panel tool variables. With deepening globalization, changes in the international market have a significant impact on China’s economic policies. In this study, we use the annual arithmetic of the global economic policy uncertainty index (Baker et al., 2016) as an instrumental variable to verify the results. The empirical research results show that economic policy uncertainty still has a significant effect on family firm innovation activities (Table 6, column 1).

**TABLE 6:** Endogenous test and robustness test.

Variable	(1)	(2)	(3)	(4)	(5)
	RD	RD	LnRD	RD	RD
EPU_*M*_	0.0456***		0.0127***		0.0476***
	(6.2180)		(7.3834)		(8.1189)
EPU				0.0211***	
				(3.9748)	
LEPU		0.0808***			
		(2.9213)			
Lev	−0.0015**	−0.0015**	−0.0000	−0.0015**	−0.0015*
	(−2.1475)	(−2.3110)	(−0.2037)	(−2.4622)	(−1.6968)
Roa	0.0322*	0.0417**	0.0129***	0.0322*	0.0958***
	(1.8765)	(1.9999)	(3.2086)	(1.6904)	(4.8956)
Tq	−0.0048***	−0.0010	−0.0007**	−0.0048***	0.0039***
	(−3.4229)	(−0.5946)	(−2.2143)	(−3.4829)	(2.6120)
Growth	0.0003**	0.0003**	0.0000	0.0003**	−0.0001
	(2.0402)	(2.0390)	(1.4635)	(2.0042)	(−0.5293)
Size	0.0267***	0.0477***	−0.0109***	0.0267***	0.0491***
	(8.4314)	(5.2685)	(−10.4812)	(4.4132)	(28.9090)
Age	−0.0159	−0.0245	−0.0037	−0.0159	−0.0149***
	(−1.4932)	(−1.1806)	(−1.2963)	(−1.0164)	(−4.2085)
Diver	−0.0021	−0.0023	−0.0002	−0.0021	−0.0013
	(−1.6248)	(−1.0278)	(−0.6543)	(−1.0878)	(−1.3829)
HHI	−0.0223	−0.0330	−0.0048	−0.0223	−0.0049
	(−1.0358)	(−0.5295)	(−0.8610)	(−0.5273)	(−0.1811)
FamHold	0.0003**	0.0002	0.0000	0.0003*	0.0003***
	(2.3619)	(0.9977)	(1.5570)	(1.7183)	(2.6313)
Seperation	−0.0029	−0.0020	−0.0025	−0.0029	0.0057
	(−0.3248)	(−0.1735)	(−1.4604)	(−0.2577)	(0.7414)
CashFlow	−0.0008	−0.0006	0.0009**	−0.0008	0.0106***
	(−0.5988)	(−0.3622)	(2.3081)	(−0.5873)	(5.9631)
_cons	−0.5952***	1.0483***	0.2341***	−0.5107***	−1.1274***
	(−4.6104)	(−5.7459)	(11.2095)	(3.9799)	(−24.7750)
var(e.RD)					0.0117***
					(52.1913)
Year	Y	Y	Y	Y	Y
Industry	Y	Y	Y	Y	Y
*N*	6469	5131	6469	6469	6469
adj. *R*^2^	0.1512	0.1390	0.1311	0.1359	0.1512

***, **, and * denote significance at 1, 5, and 10% levels.

(2) The explanatory variables lag by one period. Since economic policies are macroeconomic policies at the national level, and it is difficult for individual micro-behaviors of enterprises to influence all macroeconomic policies, there is almost no reverse causality between enterprise innovation activities and economic policy uncertainty. However, some studies find that the effect of economic policy uncertainty on enterprise activities lags behind. In this study, all explanatory variables and control variables are lagged by one period, and the fixed effects of year and industry are strictly controlled to effectively avoid the endogeneity problem of missing variables (Table 6, column 2).
(3) Variable substitution. In this study, we apply the logarithm of family business innovation investment as the explained variable of innovation investment (Table 6, column 3). Moreover, we also use the China Economic Policy Uncertainty Index (EPU) calculated by Baker et al. (2016) as an explanatory variable to study the impact of economic policy uncertainty and family business innovation activities empirically (Table 6, column 4). After the replacement, the main conclusion still holds.
(4) The regression model is extended because the enterprise innovation investment belongs to the merged data with 0 as the downline. Therefore, a Tobit model was used to test the basic hypothesis. The empirical results show that after changing the regression model, the main conclusions of this study are still valid. The specific results are shown in column 5 of Table 6.

### Additional Mechanism Test

To explore the effect of economic policy uncertainty on innovation activities, we further test the mediation role of the firm’s risk-taking capability (*Risk*_1_, *Risk*_2_), *political*, and internal capital markets (*ICM*) using the Sobel method based on the mediating mechanism model.

First, we adopt regression model (3) to test the *EPU*_*M*_ and *RD*. If the regressor is positive and α is significant, then step 2 is performed; otherwise, the test is stopped. Step 2: we adopt regression models (4) and (5) to test the intermediary effect. These show a partial mediating effect if *β_1_*, *λ_1_*, and *λ_2_* are all significant. These show a mediating effect if *β_1_* and *λ_1_* are both significant, but *λ_2_* is not significant. *Z*_*i,t*_ is a mediating variable, which take value from *Risk*_1_, *Risk*_2_, *ICM*, or *Political*.


(3) $$R\,D_{i,t}=\alpha_{0}+\alpha_{1}\,E\,P\,U_{M}+\alpha_{2}\,C\,o\,n\,t\,r\,o\,l_{i,t}+i\,n\,d\,u\,s\,t\,r\,y+y\,e\,a\,r+\varepsilon_{i,t}$$

(4) $${\text{Z}}_{i,t}=\beta_{0}+\beta_{1}\,E\,P\,U_{M}+\beta_{2}+C\,o\,n\,t\,r\,o\,l_{i,t}+i\,n\,d\,u\,s\,t\,r\,y+y\,e\,a\,r+\varepsilon_{i,t}$$

(5) $$R\,D_{i,t}=\lambda_{0}+\lambda_{1}\,E\,P\,U_{M}+\lambda_{2}+{\text{Z}}_{i,t}+C\,o\,n\,t\,r\,o\,l_{i,t}+i\,n\,d\,u\,s\,t\,r\,y+y\,e\,a\,r+\varepsilon_{i,t}$$

(1) *ICM* is used to test a family firm’s innovation activities by applying an intermediary mechanism. O’Brien (1976) studied the financing function of *ICM* by the theory of bureaucracy, which shows that integrated enterprise groups can build a capital market within an organization, facilitate financing transactions between enterprises within the group, and reduce financing costs between enterprises within the group. In this study, we follow Xie and Huang (2014) study by applying *ICM* to indicate the degree of intra-firm fund exchange. *ICM* can reduce the dependence of firm investment on cash flows and further release the pressure of firm financing. Table 7-1 shows that the regression results are 0.044 and significant at the 1% level, which means that family firms can release the pressure of firm financing under economic policy uncertainty.

**TABLE 7–1:** Internal capital market.

Variable	(1)	(2)	(3)
	RD	ICM	RD
ICM			0.0442***
			(11.4080)
EPU_*M*_	0.0005***	0.0003**	0.0210***
	(3.9223)	(2.0696)	(5.4748)
Lev	−0.0015**	0.0004	−0.0002
	(−2.4339)	(0.2163)	(−0.2122)
Roa	0.0324*	0.0725	0.0551***
	(1.7117)	(1.1843)	(3.0907)
Tq	−0.0048***	0.0071*	0.0079***
	(−3.4698)	(1.6556)	(6.5278)
CashFlow	−0.0008	−0.0018	0.0078***
	(−0.5905)	(−0.4464)	(5.3919)
Size	0.0264***	0.0259***	0.0377***
	(4.3719)	(3.5219)	(26.8153)
Age	−0.0155	−0.0236*	−0.0116***
	(−0.9927)	(−1.8533)	(−3.8876)
FamHold	0.0003*	0.0002	0.0003***
	(1.7098)	(0.8924)	(2.9681)
Seperation	−0.0029	0.0104	0.0017
	(−0.2559)	(0.7779)	(0.2742)
HHI	−0.0219	0.1328***	−0.0260**
	(−0.5175)	(2.7391)	(−2.3867)
Diver	−0.0022	0.0046	−0.0016*
	(−1.0914)	(1.0325)	(−1.8726)
Growth	0.0003**	−0.0007	−0.0002
	(2.0119)	(−1.3885)	(−0.9509)
_cons	−0.5351***	−0.5842***	−0.7714***
	(−4.2969)	(−3.5455)	(−23.7235)
Year	Y	Y	Y
Industry	Y	Y	Y
*N*	6469	6469	6469
adj. *R*^2^	0.1355		0.1679

***, **, and * denote significance at 1, 5, and 10% levels.

(2) Testing of risk-taking in family firm innovation activities building on intermediary mechanisms. Risk preference is the leading factor in perceiving decision-making scenarios and making risk decisions. Higher risk-taking means that firms show more preference toward high-risk and high-yield projects when making investment decisions, which is more conducive to maximizing the value of firms (Gao et al., 2020). The volatility of a firm’s earnings is generally used to measure risk-taking under the Chinese stock market’s high volatility. Based on John et al. (2008), we use the industry-adjusted *ROA* standard deviation *Risk*_1_ and industry-adjusted *ROA* range *Risk*_2_ to better measure the risk-taking of a family firm. Table 7-2(1) shows the Sobel test results of *Risk*_1_, and Table 7-2(2) shows the Sobel test results for *Risk*_2_. The regression results are 0.001 and 0.003, both of them are significant at the 1% level, which indicates that economic policy uncertainty can increase the risk-taking of family firms and promote the development of innovative activities.

**TABLE 7–2:** Risk-taking.

Variable	(1)	(2)	(3)
	RD	Risk_1_	RD
Risk_1_			0.001***
			(0.00)
EPU_*M*_	0.0005***	0.0059***	0.0190***
	(4.0239)	(3.4764)	(4.9368)
Lev	0.0000	0.0046	0.0001
	(0.4340)	(0.6188)	(0.8090)
Roa	0.0331*	−17.5058***	0.0698***
	(1.7238)	(−2.8468)	(3.7919)
Tq	−0.0016	0.1777***	0.0048***
	(−1.3949)	(2.6226)	(5.3293)
Growth	0.0000	0.0008	0.0000
	(1.3701)	(1.2275)	(0.4680)
Size	0.0265***	0.6439**	0.0393***
	(4.3521)	(2.5585)	(28.2275)
Age	−0.0172	−0.2528	−0.0114***
	(−1.0939)	(−0.5827)	(−3.8009)
Diver	−0.0022	0.0276	−0.0016*
	(−1.1189)	(0.3960)	(−1.8748)
HHI	−0.0214	−0.3172	−0.0248**
	(−0.5008)	(−0.3720)	(−2.2576)
FamHold	0.0003*	−0.0014	0.0003***
	(1.6931)	(−0.2349)	(2.7282)
Seperation	−0.0022	0.4118	0.0017
	(−0.1967)	(1.6207)	(0.2717)
CashFlow	−0.0009	0.0713	0.0085***
	(−0.5967)	(1.3542)	(5.8307)
_cons	−0.5481***	−9.1074*	−0.8057***
	(−4.3561)	(−1.9017)	(−25.7450)
Year	Y	Y	Y
Industry	Y	Y	Y
*N*	6469	6469	6469
adj. *R*^2^	0.1328	0.2973	0.1504

***, **, and * denote significance at 1, 5, and 10% levels.

**TABLE 7–2:** Risk-taking.

Variable	(1)	(2)	(3)
	RD	Risk_2_	RD
Risk_2_			0.0003*
			(1.8484)
EPU_*M*_	0.0001***	0.0269***	0.0212***
	(2.9574)	(8.0387)	(5.3935)
Lev	−0.0014**	−0.2453***	−0.0001
	(−2.5330)	(−3.5013)	(−0.1024)
Roa	0.0427**	−35.9769**	0.0817***
	(2.3832)	(−2.3599)	(4.4203)
Tq	0.0014	0.8320***	0.0080***
	(1.3620)	(4.6165)	(6.4056)
Growth	0.0001	0.0219	−0.0002
	(0.9711)	(1.2446)	(−1.2133)
Size	0.0421***	0.1146	0.0404***
	(11.7824)	(0.6977)	(28.6969)
Age	0.0020	−0.2257	−0.0117***
	(0.6346)	(−0.8076)	(−3.8689)
Diver	−0.0012	−0.0771	−0.0019**
	(−0.8617)	(−0.9894)	(−2.2079)
HHI	−0.0218	−2.8554	−0.0235**
	(−0.6133)	(−1.4843)	(−2.1351)
FamHold	0.0002*	0.0073	0.0003***
	(1.9341)	(0.8286)	(2.7614)
Seperation	−0.0017	0.0189	0.0024
	(−0.3037)	(0.0475)	(0.3722)
CashFlow	0.0020	0.3594**	0.0082
	(1.4118)	(2.0922)	(5.6332)
_cons	−0.9011***	6.4145	−0.8301***
	(−11.8977)	(1.3088)	(−25.4632)
Year	Y	Y	Y
Industry	Y	Y	Y
*N*	6469	6469	6469
adj. *R*^2^			0.1473

***, **, and * denote significance at 1, 5, and 10% levels.

(3) Test of the intermediary mechanism of political connections to the innovation activities of family enterprises. Due to China’s political system, business and political connections are inseparable. Interactions and connections between business and politics have received considerable attention due to the rapid growth of China in the new period. Joseph et al. (2007) argue that business and political connections do not bring positive business value after going public. On the contrary, Bian et al. (2008) argue that, instead of decreasing, business and political connections have increased. Table 7-3 shows the relationship of business and political connections with economic policy uncertainty. The regression result is −0.009, which is significant at the 1% level, which shows that by reducing political connections, family businesses reduce the cost of government intervention and rent-seeking. This reduces the ‘political cost’ of establishing and maintaining political connections, and increases the innovation input of the family business through saving the operating cost of the business.

**TABLE 7–3:** Political connections.

Variable	(1)	(2)	(3)
	RD	Political	RD
Political			−0.0092***
			(−2.8981)
EPU_*M*_	0.0002***	−0.0025***	0.0200***
	(4.6603)	(−9.1522)	(5.0984)
Lev	−0.0013**	0.0011	−0.0000
	(−2.2907)	(0.3686)	(−0.0323)
Roa	0.0488***	−0.0179	0.0598***
	(2.5971)	(−0.3159)	(3.3184)
Tq	−0.0002	−0.0179***	0.0076***
	(−0.1919)	(−3.1723)	(6.1853)
Growth	0.0001	−0.0011**	−0.0002
	(1.0283)	(−2.2385)	(−1.1485)
Size	0.0391***	0.0186**	0.0399***
	(10.7245)	(2.1261)	(28.3495)
Age	−0.0071**	−0.0356	−0.0126***
	(−2.2149)	(−1.1972)	(−4.1675)
Diver	−0.0013	0.0021	−0.0017**
	(−0.9701)	(0.4238)	(−1.9701)
HHI	−0.0063	−0.0837	−0.0233**
	(−0.1726)	(−0.8965)	(−2.1266)
FamHold	0.0002*	−0.0008	0.0003***
	(1.8770)	(−1.2572)	(2.7187)
Seperation	−0.0003	0.0314	0.0039
	(−0.0450)	(0.7459)	(0.6161)
CashFlow	0.0015	−0.0073	0.0084***
	(1.0960)	(−1.3083)	(5.7726)
_cons	−0.8419***	0.3406	−0.8120***
	(−10.9287)	(1.5576)	(−24.8839)
Year	Y	Y	Y
Industry	Y	Y	Y
*N*	6469	6469	6469
adj. *R*^2^			0.1522

***, **, and * denote significance at 1, 5, and 10% levels.

## Discussion

Between 2019 and 2020, China experienced several public emergencies, such as the United States–China trade war and the COVID-19 pandemic, both of which significantly restricted the development of firms and economy. The literature on innovation suggests that extreme shocks have the effect of firms innovate (Yang et al., 2020; Jin et al., 2021; Yonghui et al., 2021). Public emergencies can increase the cost of raw materials, reduce the employment rate, increase the pressure on enterprise operation, and cause firms to experience a crisis of survival (He et al., 2020). The extreme shocks brings a whole new level of uncertainty to the market. Altig et al. (2020) pointed out that the COVID-19 pandemic exacerbated economic policy uncertainty, with some measures of uncertainty peaking. Fernandes (2020) studied the spillover effects of the outbreak of COVID-19 on the global economy and found that COVID-19 notably increased economic policy uncertainty, which led to a global loss of public confidence. Dietrich et al. (2021) studied micro survey data from the United States and found that the level of individual responses to the uncertainty was also large (with a standard deviation of 6 to 7 percentage points), which highlighted the relationship between economic policy uncertainty and the COVID-19 pandemic. Base on the above studies, in this part, we discuss the impact of economic policy uncertainty caused by public emergencies on the innovation of family firms. The results are listed in Table 8.

**TABLE 8:** Public emergency.

Variable	(1)	(2)	(3)
	RD	RD	RD
EPU_*M*_	−0.0048*		−0.0046*
	(−1.9232)		(−1.8808)
EPU_*G*_		−0.0020*	
		(−1.9232)	
Lev	−0.0017	−0.0017	−0.0027
	(−0.6934)	(−0.6934)	(−1.1919)
Roa	0.0050	0.0050	0.0057
	(0.8819)	(0.8819)	(1.4896)
Tq	0.0016***	0.0016***	0.0016***
	(2.6280)	(2.6280)	(7.3093)
Growth	−0.0001**	−0.0001**	−0.0001**
	(−2.0597)	(−2.0597)	(−2.0011)
Size	−0.0007	−0.0007	−0.0004
	(−1.4121)	(−1.4121)	(−1.0202)
Age	−0.0032***	−0.0032***	−0.0040***
	(−3.0123)	(−3.0123)	(−3.2369)
Diver	−0.0005**	−0.0005**	−0.0004*
	(−2.5321)	(−2.5321)	(−1.8673)
CashFlow	0.0282***	0.0282***	0.0288***
	(5.0818)	(5.0818)	(5.4387)
HHI	0.0000	0.0000	0.0000
	(1.1926)	(1.1926)	(0.8883)
FamHold	−−0.0000	−0.0000	−0.0000
	(−0.1316)	(−0.1316)	(−0.1395)
Seperation	−0.0004	−0.0004	0.0006
	(−0.1839)	(−0.1839)	(0.2832)
_cons	0.0481***	0.0359***	0.0432***
	(3.3761)	(3.0103)	(3.0113)
var(e.RD)			0.0003***
			(36.4067)
Year	Y	Y	Y
Industry	Y	Y	Y
*N*	2829	2829	2829
adj. *R*^2^	0.3205	0.3205	

***, **, and * denote significance at 1, 5, and 10% levels.

Table 8 shows that, compared with the years before 2019, the economic policy uncertainty had a significant negative impact on family firm innovation in 2019–2020. Other authors who conducted similar investigations confirmed these results (Guan et al., 2021). According to Feng and Wang (2020), EPU has a strong positive effect on lower cash flow companies and companies with fewer financial constraints. Furthermore, Xu (2019) found that innovations of financially constrained firms and firms that rely on external finance in a competitive environment are affected more. In addition, from a risk-taking perspective, Guan et al. (2021) observed that a negative association between EPU and innovation mainly exists in firms whose executives have a low-risk preference and those with weak risk-taking ability. We summarize the main reasons for the economic policy uncertainty difference as follows:

(1) Before 2019, family firms could obtain sufficient financial support for innovation purposes through the internal capital market With the impact of the United States–China trade war and the COVID-2019 pandemic, however, family firms could no longer have access to such funding because of production shutdowns, cash flow disruptions, among other factors. Instead of the innovation activities, during this process, the first goal for each family firm and each family firm member is to ensure their firm’s own survival. Moreover, the intermediary, fostering innovation by enhancing internal related party transactions, was removed. This has led firms to reduce innovation investment to ensure their survival.
(2) The inheritance and protection of tacit knowledge and special assets cause family firms to take more risks when economic policy uncertainty rises. Therefore, between 2010 and 2018, family firms had more confidence in innovation. However, studies have revealed that economic policy uncertainty caused by public emergency leads to the loss of public confidence globally. Dubey (2020) collected tweets about COVID-19 from 12 countries and explored how citizens treat COVID-19 in different countries based on text mining and sentiment analysis, and found that people show negative emotions, such as anger, sadness, and disgust about COVID-19. These negative emotions reduce risk-taking, thus reducing innovation activities.

Although our findings are promising, there are still some limitations to our proposed work. First, some more advanced methods might be explored and employed to measure the economic policy uncertainty. Second, in order to ensure the integrity and continuity of economic policy uncertainty research, we employed the economic policy uncertainty index for analysis. In the future, economic policies might be classified to understand the impact of different economic policies on the innovative activities of family firms to further provide valuable suggestions for the government to formulate policies. In future studies, the team of authors will focus on the impact of COVID-19 on family firm innovation, and further investigate the central and local governments policies for private enterprise, tax, and financial support etc.

## Conclusion and Implications

### Conclusion

We examine the relationship between economic policy uncertainty and family firm innovation. Using the economic policy uncertainty index as a proxy for policy uncertainty, we find robust evidence that investment in family firm innovation is positively related to economic policy uncertainty. Moreover, this relationship is influenced by factors such as executive background and state-owned equity. Further analysis indicates that economic policy uncertainty promotes family firms’ innovation activities by improving their risk-taking ability, internal capital market circulation, and reducing political connections.

### Implications

(1) Investment in family firm innovation is positively related to economic policy uncertainty. This result indicates that stimulation by the external environment will change the objectives of the family firm and encourage enterprises to innovate positively, even though they tend to be conservative and lack innovation. Therefore, the government should focus on the development of the economy and industry, and reduce unnecessary interventions for enterprises, which revitalize private enterprises.

(2) Executives with a financial background can bring many resources for enterprises, as well as state-owned equity. This suggests that the role and effect of innovation impulses also depend on the firm’s executive characteristics and stakeholders, which are the social capital of family firms. Social capital is an important factor that affects investment in family firm innovation. To resist finance constraints, family firms should pay more attention to accumulation of social capital. Special assets can provide strong assistance for the survival and continuation of enterprises during this period. At the same time, enterprises should carry out institutional innovations to establish risk-taking funds. For enterprises, risk-taking funds can alleviate constraints on cash holdings. For the government, the financing constraints of private enterprises should be considered.

(3) Our study highlights that in family firms, economic policy uncertainty produces positive effects by reducing negative effects of political connections. Our experimental results show that family firms tend to increase their innovation investment by reducing political connections when economic policies are uncertain. There have been mixed research results on effects of political connections on firms’ innovation. First, the business environment still needs to be further improved; however, companies need to re-examine the relationship between government and enterprise. With China’s rapidly changing and reforming systems, enterprises should expand the purpose of political connection from single ‘resource extraction,’ as in the past, toward diversification of resources. Relying on rent-seeking and other methods to ask for it unrestrainedly will only reduce the legality of its existence and lead to mismatching of social resources. In the new period, the relationship between government and enterprise in system reform requires entrepreneurs to change their thinking and assume more social responsibilities. With the deepening of system transformation, political connections have placed higher demands on the role of enterprises. Entrepreneurs must change their past thinking patterns and respond effectively to the demands of various stakeholders.

## Data Availability Statement

Publicly available datasets were analyzed in this study. This data can be found here: https://www.policyuncertainty.com/china_monthly.html.

## Author Contributions

SD, YQ, SL, and SY conceived and developed the project and contributed to the interpretation of the results. SD coordinated and conducted the data collection and drafted the manuscript. SD and YQ performed the data analyses. All authors agreed to all aspects of the work and approved the final version of the manuscript.

## Conflict of Interest

The authors declare that the research was conducted in the absence of any commercial or financial relationships that could be construed as a potential conflict of interest.

## Publisher’s Note

All claims expressed in this article are solely those of the authors and do not necessarily represent those of their affiliated organizations, or those of the publisher, the editors and the reviewers. Any product that may be evaluated in this article, or claim that may be made by its manufacturer, is not guaranteed or endorsed by the publisher.

## Footnotes

1
https://www.pwc.com/gx/en/services/family-business/family-business-survey-2018.html

2 The “new normal” is a term to indicate the Chinese economy’s new growth paradigm, which is no longer characterized the high-speed growth pattern exhibited in the past. The new trend features more sustainable, mid- to high-speed growth with higher efficiency and lower costs. http://www.chinadaily.com.cn/.

3
https://www.policyuncertainty.com/china_monthly.html

4 Davis et al. (2019) figure that “People’s Daily” and “Guangming Daily” are the spokespersons of Chinese government decision makers. Therefore, they can reflect the uncertainty of the decision makers themselves. SCMP-based index shows more about the interests and views of foreign news editors on policy. Therefore, non-mainland Chinese newspapers have deviations in the concept of “uncertainty” (Qin et al., 2018).
